# Dementia risk prediction: A comparative analysis of the ANU-ADRI, CAIDE, CogDrisk, LIBRA, and LIBRA2 indices in the HUNT study

**DOI:** 10.1016/j.tjpad.2025.100326

**Published:** 2025-08-18

**Authors:** Josephine Stubs, Ellen Melbye Langballe, Gill Livingston, Kaarin J. Anstey, Kay Deckers, Fiona E. Mathews, Mika Kivimäki, Bjørn Heine Strand, Anne-Marie Rokstad, Steinar Krokstad, Geir Selbæk

**Affiliations:** a Norwegian National Centre for Ageing and Health, Vestfold Hospital Trust, Aldring og helse, 103 Tønsberg, Norway; b Department of Geriatric Medicine, Oslo University Hospital, Kirkeveien 166, 0450 Oslo Norway; c Faculty of Medicine, Institute of Clinical Medicine, University of Oslo, Kirkeveien 166, 0450 Oslo, Norway; d Division of Psychiatry, 149 Tottenham Court Rd, University College London, London, W1W 7EJ UK; e North London NHS Foundation Trust, St Pancras Hospital, London, NW1 0PE UK; f School of Psychology, University of New South Wales, Sydney, Australia; g Neuroscience Research Australia, Sydney, Australia; h UNSW Ageing Futures Institute, University of New South Wales, Sydney, Australia; i Alzheimer Centrum Limburg, Department of Psychiatry and Neuropsychology, Mental Health and Neuroscience Research Institute (MHeNs), Maastricht University, Dr. Tanslaan 12, 6229 ET, Maastricht, The Netherlands; j Institute for Clinical and Applied Health Research, University of Hull, Cottingham Road, Hull, HU6 7RX, UK; k Department of Public Health, Faculty of Medicine, University of Helsinki, Tukholmankatu 8 B, FI-00014 University of Helsinki, Finland; l Department of Physical Health and Ageing, Norwegian Institute of Public Health, Oslo, Norway; m Faculty of Health Sciences and Social Care, Molde University College, Postboks 2110, 6402 Molde, Norway; n Department of Public Health and Nursing, Faculty of Medicine and Health Sciences, HUNT Research Centre, Norwegian University of Science and Technology, Trondheim, Norway; o Levanger Hospital, Nord-Trøndelag Hospital Trust, Levanger, Norway

**Keywords:** Dementia, Risk index, Modifiable risk factors, HUNT, Lifestyle

## Abstract

**Background/Objective:**

Dementia is a major global health concern, necessitating effective risk assessment tools and early intervention. This study compared the performance of five modifiable dementia risk indices – ANU-ADRI, CAIDE, CogDrisk, LIBRA, and LIBRA2 and a “demographics-only” (age, education) model.

**Methods:**

We analyzed data from 5247 Norwegian participants in the Trøndelag Health Study (HUNT4 70+, 2017–2019) and dementia risk indices from baseline data in HUNT3 (2006–2008). Logistic regression models assessed associations between standardized index scores and all-cause dementia and Alzheimer’s disease (AD) across age group (<65 vs. ≥65 years), sex, and APOE4 status.

**Results:**

During the mean follow-up of 10.6 (9.3–12.3) years (SD=0.74), all indices significantly predicted dementia and AD, though none outperformed the demographics-only model. CogDrisk showed significantly better discriminative ability than all other indices (0.76, 95 % CI:0.74–0.78; DeLong *p* < 0.05), followed by LIBRA (0.75, 95 % CI:0.72–0.77) and ANU-ADRI (0.74, 95 % CI:0.72–0.76). LIBRA2 (0.69, 95 % CI:0.66–0.71) and CAIDE (0.59, 95 % CI:0.56–0.61) had significantly lower accuracy (DeLong *p* < 0.001). Removing demographics maintained rank order but reduced accuracy across all indices. Stratified analyses showed stronger performance in those ≥65 years and females at HUNT3, while APOE4 status did not affect performance.

**Conclusion:**

All indices were associated with dementia risk, with CogDrisk performing best across all conditions, and LIBRA2 and CAIDE performing weakest. No index outperformed a model including age and education only. Future research should refine risk indices for age- and sex-specific applications and assess whether simpler demographic models may suffice in some contexts.

## Highlights

• 5 dementia risk scores and a demographics-only model were compared longitudinally.
• CogDrisk and LIBRA showed the best overall accuracy in predicting dementia risk.
• ANU-ADRI showed moderate predictive performance.
• CAIDE and LIBRA2 performed weakest across models and subgroups.
• No index outperformed age and education alone in predicting dementia.

## 1 Introduction

Dementia is a major global health challenge, affecting over 57 million people worldwide, with prevalence projected to rise to 153 million by 2050 [1]. Beyond its devastating personal and societal cost, dementia presents a serious economic burden, with global costs exceeding $1 trillion annually [2]. Delaying dementia onset by just five years is estimated to reduce its incidence by 33 % [3]. Furthermore, increasing the years individuals live independently or with low support needs, would substantially alleviate both financial and emotional pressures on individuals, families, and healthcare systems [3]. This may be possible through improvements in risk reduction, intervention, and early identification of dementia pathology – avenues that have seen large strides in recent years, for example with new low-cost and minimally invasive diagnostic tests [4,5], increased knowledge on modifiable dementia risk factors [6], and potentially emerging anti-amyloid antibody treatments [[7], [8], [9]].

The Lancet Commission on Dementia Prevention, Intervention, and Care has estimated that 14 modifiable risk factors account for roughly 45 % of dementia risk [6]. This finding underscores the potential for targeted lifestyle interventions to reduce the global dementia burden and can be tackled jointly by individuals, physicians, researchers, and policymakers. Multiple tools have been developed and validated over the past decade, enabling the mapping of modifiable dementia risk and protective factors in individuals and populations. Among the most well-studied are the Australian National University Alzheimer’s Disease Risk Index (ANU-ADRI) [10], the Cardiovascular Risk Factors, Aging, and Dementia Study (CAIDE) [11], the LIfestyle for BRAin Health (LIBRA) index [12] and its updated version (LIBRA2) [13], and the more recently developed CogDrisk Index [14]. These indices focus on modifiable risk factors, which are combined into a single dementia risk score.

While most of these indices were originally developed to support prevention and user guidance, they have the potential to play a crucial role in a collaborative approach to dementia risk reduction and are increasingly used across a broad spectrum of applications. For individuals, they highlight modifiable risk factors and enable early lifestyle changes, as well as low-cost, accessible self-assessment – especially for those who lack regular health care accessibility. Each index now has an app or website allowing individuals to self-assess at home [15]. Physicians may use them to stratify risk, guide personalized risk reduction plans, and improve communication about risk with patients. Researchers benefit from standardized frameworks for measuring risk, selecting high-risk participants for trials, and using indices as surrogate outcomes when direct dementia diagnoses are impractical [15]. Policymakers can benefit from aggregated data to identify high-risk populations, design targeted public health initiatives and monitor intervention impact over time.

Despite their potential, further studies are needed to assess their validity and limitations in different contexts to ensure appropriate application. Given their resource-intensive computational and data collection demands, thorough validation and optimization are crucial to avoid placing an undue burden on overtaxed healthcare systems or imposing computational strain in research and clinical settings, particularly in contexts where simpler models, such as those based on age, sex, and education, may suffice. While each index has been tested and validated across diverse cohorts, results show varying predictive performance [13,[16], [17], [18], [19], [20], [21], [22], [23], [24], [25], [26], [27]]. ANU-ADRI, developed through evidence synthesis, has shown low-to-moderate accuracy (AUC=0.52–0.75) [24,26,28], performing well in the Rotterdam Study (AUC=0.75) [16] but less so in the UK Biobank (AUC=0–58–0.59) [27,28] which has a younger age range than many cohorts. CAIDE, derived from Finnish population data, has demonstrated strong performance in studies with extended follow-ups, achieving AUCs as high as 0.78 [11,16], but its accuracy is lower in other cohorts (AUC=0.50–0.68) [24,26,28,29]. LIBRA, developed through evidence synthesis focused on midlife modifiable risk and protective factors, shows low to moderate accuracy (AUC=0.52–0.75) [30], and is less reliable in very old cohorts (75+ years) [31]. However, comparability between different studies is problematic as some studies include age as a covariate while others do not. Recently developed CogDrisk performs with moderate accuracy across populations, with AUCs ranging from 0.66 to 0.77 [24]. Notably, age alone often performs as well as or better than full indices [32], underscoring the challenge of improving prediction models beyond demographic factors such as age, gender, and education.

Most risk factors in these indices are well-validated individually and offer value for users assessing how their lifestyle affects dementia risk. However, their growing use in research and other settings necessitates assessing their effectiveness beyond their original intent. Moreover, the growing number of indices calls for a comprehensive comparison of their performance in different contexts. Particularly, subgroup comparisons based on age, sex, and genetic risk (Apolipoprotein epsilon 4 allele (APOE4) status) are warranted, as these variables are known to moderate the association between lifestyle factors and dementia risk [6,[32], [33], [34]]. Despite widespread use of these indices individually, directly comparing their predictive performance within the same cohort remains rare. Only one other study to date has compared all five of the most commonly used dementia risk indices [24]. Therefore, this study aims to compare the predictive performance of these dementia risk indices (ANU-ADRI, CAIDE, CogDrisk, LIBRA and LIBRA2) against each other and a simpler demographic model (age and education), across all eligible participants of the Trøndelag Health Study (HUNT) and within subgroups stratified by age, self-reported sex, and APOE4 status.

## 2 Method

### 2.1 Study population

This study used data from the HUNT Study, a population-based cohort in Trøndelag, Norway, ongoing since 1984. HUNT includes four surveys conducted every 10–11 years, collecting health, lifestyle, and disease data via clinical exams, interviews, questionnaires, and biological samples [35]. Participants also consented to data linkage with national health registries. Beyond informed consent given by HUNT participants, the Regional Committee for Medical and Health Research Ethics in Norway (REK Southeast 251,687) and the Norwegian Center for Research Data (NSD 571,736), evaluated and approved this project and data linkage between the different registries.

This study focused on participants from HUNT4 70+, a sub-study of the fourth HUNT survey (HUNT4, 2017–2019) targeting individuals aged 70 and older. Lifestyle and health data for these participants were collected about 11 years earlier during HUNT3 (2006–2008), enabling longitudinal risk factor analysis. HUNT3 was selected as baseline because it includes nearly all variables used in the dementia risk indices and aligns with the start of available registry data from the Norwegian Patient Registry (NPR) in 2007. Additional linkage to data from Statistics Norway (SSB) and NPR provided access to education records and disease diagnoses (3-digit ICD-10 codes, recorded in specialized health care settings) between 2007 and 2009. Of 9930 individuals who participated in HUNT4 70+ (representing 51.2 % of the local population aged 70 and older in Trøndelag [36]), 9726 (98.0 %) provided sufficient information to inform dementia status and constituted the eligible follow-up sample for outcome classification in this study. Among these, 8397 (86.3 %) had also participated in HUNT3 and were eligible for risk score calculation. The main analysis excluded participants with incomplete risk score data (*n* = 3268, 32.9 %) that could not be supplemented with registry data (NPR/SSB), yielding a final analytical sample of 5247 individuals.

### 2.2 Materials

We compared the predictive performance of ANU-ADRI, CAIDE, CogDrisk, and LIBRA, and LIBRA2. Table 1 provides the variables and weights included in these indices. ANU-ADRI was developed to estimate AD risk in public health settings for adults 65 and older [10]. It developed through literature reviews and meta-analyses identifying 11 risk factors, and 4 protective factors associated with AD. Risk ratios were converted into point-based scores, and summed to provide individual risk scores. This index includes pesticide exposure, which has been excluded in this study, due to limited data availability. CAIDE, focuses on midlife risk assessment and was developed in a Finnish cohort aged 39 to 64 years, followed for 20 years to assess dementia outcomes [11]. It incorporates vascular risk factors, age, sex, education, and APOE4 status. The score derived using logistic regression coefficients from baseline risk factors. CogDrisk assess dementia risk in adults aged 65 years and older, incorporating 17 risk factors identified through systematic reviews and meta-analyses [14]. Risk ratios for each factor were converted into points and summed to create an individual risk score. LIBRA, now validated in over 25 cohorts [21], was developed based on a systematic review and Delphi consensus, focusing on twelve modifiable dementia risk factors such as diet, physical activity, cardiovascular-, and psychosocial risks [12]. Relative risk estimates from meta-analyses were used to derive weights which are summed to yield a total score. LIBRA2, a recently modified version, incorporates three additional variables (sleep, social contact, and hearing impairment) [13]. Neither version includes non-modifiable factors (such as age, sex, and education), reflecting their focus on lifestyle-based risk reduction. However, to aide comparability to other indices, previous studies have commonly included these variables in the index [37]. In line with previous studies [24], the current study includes age, sex, and education into the LIBRA score using ANU-ADRI’s scoring. Additionally, this study also compared all indices without age, sex, and education. The LIBRA Index includes chronic kidney disease; however, this item was omitted in this study as this data was not accessible.

**Table 1:** Descriptive table displaying all variables and their weights included in each index, as well profile of included and missing population.

Variable	Level	N included	N missing	ANU-ADRI	CAIDE	CogDrisk	LIBRA	LIBRA2
**Sex**	Women	2768 (52.8 %)	2525 (56.4 %) ***	–	0	–	–	–
	Men	2479 (47.2 %)	1954 (43.6 %) ***	–	1	–	–	–
**Age Baseline (HUNT3)**	57.8–90.0; M:66.6 (SD: 5.8)							
**Age Follow-up (HUNT4 70+)**	70.0–100.4; M:77.2 (SD: 5.8)							
**Dementia**	No Dementia	4681 (89.2 %)	3538 (79.0 %)***					
	All Type Dementia	566 (10.8 %)	941 (21.0 %)***					
**Age (Males)**	<47	0 (0.0 %)	0 (0.0 %)	–	0	–	–	–
	47–53	0 (0.0 %)	0 (0.0 %)	–	3	–	–	–
	>53	2479 (100 %)	1316 (100 %)	–	5	–	–	–
**Age (Males)**	<65	1251 (50.5 %)	546 (41.5 %)***	0	–	0	(0)^a^	(0) a
	65–69	651 (26.3 %)	342 (26.0 %)***	1	–	6	(0.4)	(0.4)
	70–74	366 (14.8 %)	230 (17.5 %)***	12	–	8	(5.2)	(5.2)
	75–79	143 (5.8 %)	136 (10.3 %)***	18	–	13	(6.8)	(6.8)
	80–84	61 (2.5 %)	53 (4.0 %)***	26	–	17	(11.2)	(11.2)
	85–89	7 (0.3 %)	9 (0.7 %)***	33	–	20	(14.1)	(14.1)
	>90	0 (0.0 %)	0 (0.0 %)	38	–	22	(16.4)	(16.4)
**Age (Females)**	<47	0 (0.0 %)	0 (0.0 %)	–	0	–	–	–
	47–53	0 (0.0 %)	0 (0.0 %)	–	3	–	–	–
	>53	2768 (100 %)	1831 (100 %)	–	5	–	–	–
**Age (Females)**	<65	1318 (47.6 %)	689 (37.6 %)***	0	–	0	(0)	(0)
	65–69	699 (25.2 %)	453 (24.8 %)***	5	–	4	(2.1)	(2.1)
	70–74	421 (15.2 %)	307 (16.8 %)***	14	–	7	(6.2)	(6.2)
	75–79	239 (8.6 %)	230 (12.6 %)***	21	–	11	(9.2)	(9.2)
	80–84	67 (2.4 %)	127 (6.9 %)***	29	–	15	(12.4)	(12.4)
	85–89	23 (0.8 %)	21 (1.2 %)***	35	–	19	(15.3)	(15.3)
	>90	1 (0.04 %)	4 (0.2 %)***	41	–	23	(17.6)	(17.6)
**Education**	<8 years / Low	3 (0.06 %)	2 (0.04 %)***	0	4	4	(2.7)	(2.7)
	8–11 years / Medium	3376 (64.3 %)	3172 (70.9 %)***	3	3	2	(1.4)	(1.4)
	>11 years / High	1868 (35.6 %)	1305 (29.1 %)***	6	0	0	(0)	(0)
**Alcohol Use**	None	3924 (74.8 %)	1710 (69.9 %)***	0	–	–	0	0
	Light–Moderate (1–14 units/wk)	1295 (24.7 %)	711 (29.1 %)***	−3	–	–	−1	0
	Excessive (≥14 units/wk)	28 (0.5 %)	26 (1.1 %)***	0	–	–	0	3.1
**APOE ε4 Carrier**	Non-carrier	3848 (73.3 %)	3183 (72.7 %)	–	0	–	–	–
	Carrier^b^	1399 (26.7 %)	1195 (27.3 %)	–	2	–	–	–
**BMI**	Underweight (<18.5)	21 (0.4 %)	9 (0.3 %)	–	0	2	–	–
	Normal (18.5–24.99)	1272 (24.2 %)	780 (25.1 %)	0	0	0	0	0
	Overweight (25–29.99)	2662 (50.7 %)	1545 (49.7 %)	2	0	1	0	0
	Obese (≥30)	1292 (24.6 %)	777 (25.0 %)	5	2	3	1.6	7
**Cognitive Activity**	Low	1428 (27.2 %)	941 (42.6 %)***	0	–	0	0	9.4
	Medium	1874 (35.7 %)	743 (33.7 %)***	−6	–	–4	0	0
	High	1945 (37.1 %)	524 (23.7 %)***	−7	–	–5	−3.2	0
**Coronary Heart Disease**	No	4376 (83.4 %)	3802 (84.9 %)*	–	–	0	–	–
	Yes	871 (16.6 %)	677 (15.1 %)*	–	–	2	1	8.3
**Depression**	No	4733 (90.2 %)	4224 (94.3 %)***	0	–	0	0	0
	Yes	514 (9.8 %)	255 (5.7 %)***	2	–	3	2.1	13
**Diabetes**	No	4804 (91.6 %)	4040 (90.2 %)*	0	–	0 (M), 0 (F)	0	0
	Yes	443 (8.4 %)	439 (9.8 %)*	3	–	2 (M), 3 (F)	1.3	6.8
**Diet: Fish Intake**	0–0.25 servings/week	1205 (23.0 %)	569 (19.6 %)***	0	–	0	–	–
	0.26–2 servings/week	3600 (68.6 %)	1993 (68.7 %)***	−3	–	−0.25	–	–
	2.1–4 servings/week	321 (6.1 %)	245 (8.5 %)***	−4	–	−0.25	–	–
	>4.1 servings/week	121 (2.3 %)	93 (3.2 %)***	−5	–	−0.25	–	–
**Diet: Mediterranean**	Yes	3031 (57.8 %)	1621 (55.9 %)	–	–	–	−1.7	0
	No	2216 (42.2 %)	1278 (44.1 %)	–	–	–	0	3.8
**Hearing Impairment**	No	2882 (54.9 %)	2795 (62.4 %)***	–	–	–	–	0
	Yes	2365 (45.1 %)	1684 (37.6 %)***	–	–	–	–	7.6
**Hypercholesterolemia**	No	3928 (74.9 %)	2213 (74.8 %)	0	0	0	0	0
	Yes	1319 (25.1 %)	746 (25.2 %)	3	1	3	1.4	8.2
**Hypertension**	No (Systolic <140 mm Hg)	2788 (53.1 %)	1624 (36.3 %)***	–	0	0	0	0
	Yes (Systolic ≥140 mm Hg)	2459 (46.9 %)	2855 (63.7 %)***	–	2	1	1.6	3.5
**Physical Activity**	Low	943 (18.0 %)	464 (24.8 %)***	0	1	0	1.1	6
	Medium	42 (0.8 %)	23 (1.2 %)***	−2	0	−3	0	0
	High	4262 (81.2 %)	1388 (74.0 %)***	−3	0	−3	0	0
**Traumatic Brain Injury**	No	4906 (93.5 %)	4201 (93.8 %)	0	–	0	–	–
	Yes	341 (6.5 %)	278 (6.2 %)	4	–	2	–	–
**Sleep Problems**	No	3590 (68.4 %)	1472 (67.4 %)	–	–	0	–	0
	Yes	1657 (31.6 %)	713 (32.6 %)	–	–	2	–	3.3
**Smoking**	No	2216 (42.2 %)	1197 (41.7 %)	0	–	0	0	0
	Former Smoker	2224 (42.4 %)	1179 (41.1 %)	1	–	–	–	–
	Yes	807 (15.4 %)	492 (17.2 %)	4	–	1	1.5	7.9
**Social Activity**	Lowest	1245 (23.7 %)	604 (25.0 %)	0	–	–	–	0
	Low to Medium	1344 (25.6 %)	576 (23.9 %)	1	–	–	–	0
	Medium to High	1344 (25.6 %)	630 (26.1 %)	4	–	–	–	0
	Highest	1314 (25.0 %)	602 (25.0 %)	6	–	–	–	6.5
**Social: Loneliness**	Not Lonely	4302 (82.0 %)	2002 (79.4 %)**	–	–	0	–	–
	Lonely	945 (18.0 %)	521 (80.7 %)**	–	–	2	–	–
**Stroke History**	No	5044 (96.1 %)	3011 (95.8 %)	–	–	0	–	–
	Yes	203 (3.9 %)	132 (4.2 %)	–	–	2	–	–
**Pesticide Exposure**^c^	Never	–	–	0	–	–	–	–
	Ever	–	–	2	–	–	–	–
**Chronic Kidney Disease**^c^	Yes	–	–	–	–	–	1.1	5.7
	No	–	–	–	–	–	0	0
**Total Score (min - max; M; (SD))**			−14–53; M: 5.9 (SD: 10.0)	5.0–16.0; M:9.2 (SD: 2.0)	−8.25–31.75; M:3.5 (SD: 7.0)	−5.9–23.1; M:3.0 (SD: 5.0)	0 – 82.5; M:29.1 (SD: 13.7)	

Notes: Chi² tests assessed differences in variable distributions between included and excluded participants. Missing refers to participants eligible for dementia classification but excluded from main analysis due to missing HUNT3 data or incomplete risk factor information. Significance levels: *** *p* < 0.001, ** *p* < 0.005, * *p* < 0.05.

a LIBRA and LIBRA2 were originally developed without assigning points for age, and education, therefore these variables are in parentheses. To aid comparability many studies, including this one have assigned age and education weights.

b APOE4 Carrier: heterozygotes=1261, homozygotes=138.

c Pesticide exposure and chronic kidney disease are omitted from all analyses as this data was not available.

### 2.3 Data preparation and index generation

All variables were derived from participant responses and measurements collected during HUNT3 (2006–2008), and supplemented by HUNT1 (1984–1986), HUNT2 (1995–1997), SSB (2007) and NPR (2007–2009) to address missing data. All data were harmonized and recoded to align with the definitions and requirements of each risk index. This harmonization ensured variables across indices were consistently defined, categorized, and scored according to their respective scoring systems (see Table 1), enabling meaningful comparisons.

#### 2.3.1 Dementia status

Dementia diagnoses in HUNT4 70+ were established by a Diagnostic Consensus Committee of nine medical doctors with expertise in geriatrics, neurology, and old-age psychiatry, combining clinical and research experience [38]. Diagnoses followed Diagnostic and Statistical Manual of Mental Disorders, Fifth Edition (DSM-5) criteria, classifying participants into one of the following categories: (1) no cognitive impairment, (2) amnestic- and (3) nonamnestic mild cognitive impairment, and (4) dementia. Participants with dementia were further categorized as having AD or other dementia types (see [35]). In this study, dementia was coded as “No Dementia” (no or mild cognitive impairment) and “Dementia” (any dementia diagnosis). We also ran supplementary analyses including AD status only, defined as “no dementia” vs. “AD,” with other dementias coded as missing.

#### 2.3.2 Education

Education was obtained from Statistics Norway (SSB, 2007) and categorized into three levels primary, secondary, tertiary (<8 years, 8–12 years, and >12 years of formal education) [10,14,24].

#### 2.3.3 Alcohol consumption

Alcohol intake was based on HUNT3 self-reported weekly units and categorized as: none (0 units/week), low/moderate (1–14), and high (≥14) [10,30].

#### 2.3.4 APOE4 status

DNA from HUNT participants was analyzed using Illumina HumanCoreExome arrays. APOE genotyping used rs429358 and rs7412 single nucleotide polymorphisms to determine ε4 allele presence [39]. Following CAIDE protocol, participants were classified as having “no ε4 allele” or “at least one ε4 allele.”

#### 2.3.5 Body mass index (BMI)

BMI was calculated using the standard formula (kg/m²), based on participants’ weight (in kilograms, rounded to one decimal) and height (in meters, rounded to two decimals). Measurements were taken on the HUNT3 participation day, with participants wearing light clothing and no shoes. BMI was categorized as underweight (<18.5), normal weight (18.5–24.99), overweight (25–29.99), and obese (≥30) [40].

#### 2.3.6 Cognitive activity

Cognitive activity was assessed using a composite score from self-reported work and leisure activities selected for closely resembling questions included in ANU-ADRI and CogDrisk [10,14]. These included hours using a computer at work, computer use for leisure, and frequency of attending museums, concerts, or participating in music and theatre. The combined score was grouped into tertiles: low, medium, and high cognitive activity.

#### 2.3.7 Cholesterol

Serum total cholesterol levels were obtained from non-fasting blood samples taken at HUNT3 and categorized as normal (<6.5 mmol/L) or high (≥6.5 mmol/L) [11].

#### 2.3.8 Depression

Depression was measured using the Hospital Anxiety and Depression Scale [41], with scores ≥8 classified as depression [41]. Where HUNT3 data were missing, NPR data (ICD10 F32–33) was used for participants who had been diagnosed with depression by their health care provider between 2007 and 2009.

#### 2.3.9 Diabetes

Diabetes status was determined using self-reported diagnoses or non-fasting glucose levels (≥9 mmol/L) from HUNT3. HUNT3 data were validated and, if missing, supplemented using earlier HUNT surveys and NPR (ICD10 E10–E14). A self-reported diabetes diagnosis or elevated non-fasting glucose levels (≥9 mmol/L) [42] were considered a risk factor.

#### 2.3.10 Fish consumption and healthy diet

Fish consumption was self-reported in HUNT3 and categorized into four groups: 0–3 times/month, 1–3 times/week, 4–6 times/week, and ≥1 time/day. A healthy diet score was based on the frequency of fruit, vegetable, and fish consumption. Points from 1 to 4 (1=“0–3 times/month”, 4=“every day or more”) were assigned to each food group, summed, and classified as healthy (≥10 points) or unhealthy (<10) [10,13,14].

#### 2.3.11 Hearing loss

Hearing loss was determined using self-reported data from HUNT1, 2, and 3. Participants were asked if they had hearing loss/impaired hearing they were aware of (yes/no) [12,13]. Hearing loss in HUNT1 or HUNT2 was carried forward to HUNT3.

#### 2.3.12 Heart disease

The LIBRA index includes coronary heart disease [12,13], while CogDrisk [14] includes arterial fibrillation. A proxy variable for heart disease was created, incorporating diagnoses of angina pectoris, heart failure, myocardial infarction, and other specified heart diseases, to capture participants with potential cardiovascular issues. Participants reporting any of these conditions were categorized as having heart disease. NPR data (ICD10 I11, I20–I25, I50–I51) supplemented missing HUNT3 data.

#### 2.3.13 Hypertension

Blood pressure was measured in a seated position following standardized protocols at HUNT3. After two minutes of rest, three automatic oscillometric readings were taken at one-minute intervals, with the mean of the second and third used in analyses. Systolic and diastolic pressures were recorded to the nearest 2 mm Hg. In line with all indices, hypertension was defined as a mean systolic blood pressure ≥140 mm Hg [[11], [12], [13]]. If HUNT3 data were missing, NPR diagnoses (ICD10 I10, I15) were used to classify participants as hypertensive.

#### 2.3.14 Physical activity

Physical activity was measured as total weekly minutes of moderate-to-vigorous physical activity (MVPA) based on self-reported levels in HUNT3. Participants engaging in ≥150 min per week were categorized as “highly active,” 30–149 min as “medium active,” and ≤29 min as “inactive” in the ANU-ADRI [10], CogDrisk [14], and LIBRA [12,13] indices. Instead of three levels, the CAIDE Index used a dichotomous variable (active/inactive). In line with CAIDE’s scoring criteria [11], “active” was defined as at least 30 min, at least twice per week.

#### 2.3.15 Smoking status

Self-reported smoking status at HUNT3 was categorized into three groups: current smoker, ex-smoker, and non-smoker. Current smoking was consistently classified as a risk factor across all indices [[11], [12], [13], [14]]. While ANU-ADRI assigned risk points to ex-smokers [10], the other indices grouped them with non-smokers into a single category.

#### 2.3.16 Social activity

In line with cognitive activity, social activity was assessed using a composite score derived from multiple self-reported social life measures, selected to imitate the ANU-ADRI [10] survey as closely as HUNT3 data allowed. ANU-ADRI considers five factors: (1) marital status, (2) social network size, (3) social network quality, (4) level of social activities, and (5) living arrangements. Marital status and living arrangements were based on whether participants lived with a spouse or domestic partner (yes/no). Social network quality was evaluated using responses to: "Has friends who provide help" and "Has friends to talk to confidentially" (yes/no). Participants also rated agreement with: "I feel a strong sense of community with the people who live here," "We trust each other here," and "People like living here," on a 1 (strongly disagree) to 5 (strongly agree) scale. Level of social activity was measured by frequency of participation in social activities such as associations or church services, rated from 1 (never) to 5 (very frequent). A composite score was created by standardizing and aggregating these measures, then dividing participants into quartiles: low, low-medium, high-medium, and high. The lowest quartile was also categorized as "low social activity" in the LIBRA index. CogDrisk measures social activity as “lonely”, “not lonely” [14] which is a survey question in HUNT and could be used directly by coding participants who reported some to strong loneliness as “lonely” and others as “not lonely”.

#### 2.3.17 Sleep disturbance

HUNT3 sleep disturbance was defined as experiencing difficulties falling asleep, staying asleep, or waking early “several times a week.” Participants reporting frequent disturbances of at least one category were categorized as having sleep disturbance [13].

#### 2.3.18 Stroke

Stroke history was determined using self-reported data (yes/no) [14] from HUNT3, supplemented with data from NPR (ICD10 I60–I69) and earlier HUNT surveys if stroke data for HUNT3 was missing.

#### 2.3.19 Traumatic brain injury (TBI)

TBI was assessed using self-reported data from HUNT2, asking whether participants had ever been hospitalized for a head injury [10,14]. Additionally, data from NPR (ICD10 S06–S07, S09) was used to account for participants treated for head injuries in the years 2007–2009.

### 2.4 Statistical analysis

Stata 18 (StataCorp) was used for all analyses. Descriptive statistics summarized baseline characteristics and examined missing data for the dementia risk indices. Differences between participants with complete and incomplete data were assessed using chi-squared tests, with statistical significance set at *p* < 0.05 .

Logistic regression models were employed to evaluate the association between each risk index and dementia status (dementia vs. no dementia), and AD status (AD vs. no dementia). All indices were analyzed in two forms: (i) including age, sex, and education weights, and (ii) excluding them. This was done to compare both the complete indices like they are commonly used, and to compare just the lifestyle risk factors aspects of each index. Additionally, this aided in comparison with the LIBRA indices, which were originally developed without demographic variables. Though similar in distributional shape, the original indices varied substantially in scale and dispersion (Table 1). To account for these differences and facilitate comparison across indices with varying scoring systems, all risk indices were standardized to z-scores (Mean=0, SD=1) prior to analysis. Additional logistic regression models were run to predict dementia using (i) age, sex, and education, and (ii) age and education. Sex did not significantly contribute to the model (*p* = 0.827) and was therefore excluded from further analyses. The final demographics only model (age and education) was used as the reference model for evaluating the predictive performance of the risk indices using the receiver operating characteristic curve (AUC). To enable meaningful comparison of predictive performance and statistical AUC testing, all primary analyses were conducted on the same sample of participants with complete data across all indices. To minimize the inflation of Type I error from multiple comparisons, we first conducted a global DeLong test [43] comparing the AUCs of all indices. Only if the global comparison reached statistical significance (*p* ≤ 0.05) did we proceed with pairwise ROC comparisons between indices. AUC values were interpreted using standard thresholds: 0.5–0.6=“fail”, 0.6–0.7=“poor”, 0.7–0.8=“moderate”, and 0.8–0.9=“good” [44]. Finally, we conducted stratified analyses by sex (male/female), baseline age group (≤65 vs. >65), and APOE4 status (non-carrier vs. at least one ε4 allele).

#### 2.4.1 Sensitivity analysis

To address missing data and evaluate the robustness of our findings, sensitivity analyses were conducted using joint multivariate normal imputation [45]. A total of 100 imputed datasets were generated using age, sex, education, and dementia status as predictors. Logistic regression models were run within each imputed dataset, and pooled estimates were derived using Rubin’s rules. The imputed results were compared with the complete case analysis, focusing on coefficients, significance levels, Relative Variance Increase (RVI) and Fraction of Missing Information (FMI). Additionally, to assess whether restricting the main analyses to complete cases influenced findings, we reran each index-specific model using all participants with available data for that specific index, regardless of missingness in other indices.

## 3 Results

### 3.1 Summary statistics

The study included 5247 participants (52.8 % women), with a mean age of 66.6 years (SD=5.8, range: 57.8–90.0) at baseline (HUNT3), and 77.2 years (SD=5.8, range: 70.0–100.4) at follow-up (HUNT4). Dementia was present in 10.8 % of participants, with AD (6.3 %) being the most prevalent subtype (Table 1).

Participants with missing data were significantly older, more often female, and had lower educational attainment. Dementia, diabetes, hypertension, physical inactivity, and smoking were significantly more prevalent in this group, while depression was significantly less common. No significant differences were found for coronary heart disease, BMI, or sleep problems (Table 1).

### 3.2 Dementia risk indices

**Fig. 1:**
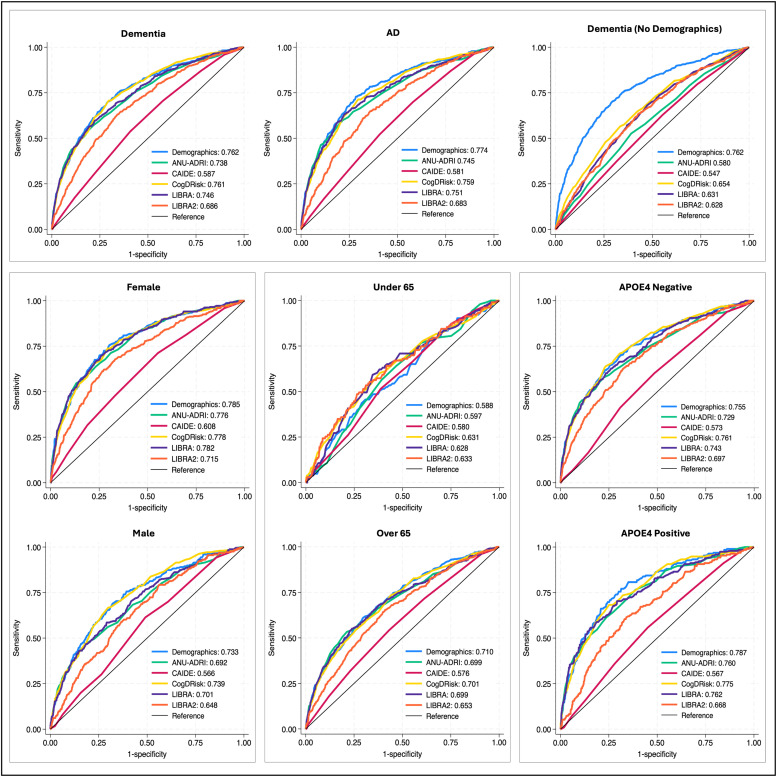
ROC curves for all indices under different conditions. Notes: ROC curves for models including age and education weighting (see Table 3 for full model info) Legends display AUC values for each curve.

All dementia risk indices were associated with dementia status (Table 2). CogDrisk showed the strongest association (OR=2.57, 95 %CI: 2.35–2.81) and the highest discriminatory ability (AUC=0.761), followed by LIBRA (OR=2.51, 95 %CI: 2.30–2.74; AUC=0.746; Table 3) and ANU-ADRI (OR=2.39, 95 %CI: 2.20–2.60; AUC=0.738). LIBRA2 demonstrated moderate performance (OR=1.93, 95 %CI: 1.77–2.11; AUC=0.686), while CAIDE had the weakest predictive ability (OR=1.35, 95 %CI: 1.24–1.47; AUC=0.587). However, none of the indices outperformed a demographics-only model (age, education; AUC=0.762; DeLong’s *p* > 0.05). Moreover, ANU-ADRI (*p* = 0.015), CAIDE (*p* < 0.001), and LIBRA2 (*p* < 0.001) performed significantly worse (Fig. 1).

**Table 2:** Table showing all logistic regression models, for ANU-ADRI, CAIDE, CogDrisk, LIBRA, LIBRA2, and a demographics only model.

	ANU-ADRI		CAIDE		CogDrisk		LIBRA		LIBRA 2		Demographics		
Model	Odds Ratio (95 %CI)	Pseudo r-squared	Odds Ratio (95 %CI)	Pseudo r-squared	Odds Ratio (95 %CI)	Pseudo r-squared	Odds Ratio (95 %CI)	Pseudo r-squared	Odds Ratio (95 %CI)	Pseudo r-squared	Variable	Odds Ratio	Pseudo r-squared
**Dementia**	2.39 (2.20, 2.60)***	0.13***	1.35 (1.24, 1.47)***	0.01***	2.57 (2.35, 2.81)***	0.14***	2.51 (2.30, 2.74)***	0.13***	1.93 (1.77, 2.11)***	0.06***	**Age**	1.16 (1.14, 1.18)***	0.14***
											**Education**	0.62 (0.50, 0.78)***	
**Dementia No Demographics**	1.32 (1.21, 1.44)***	0.01***	1.17 (1.08, 1.28)***	0.00***	1.72 (1.58, 1.87)***	0.05***	1.59 (1.45, 1.74)***	0.03***	1.54 (1.41, 1.68)***	0.03***			
		
**AD**	2.45 (2.21, 2.72)***	0.12***	1.33 (1.19, 1.48)***	0.01***	2.55 (2.29, 2.85)***	0.12***	2.56 (2.29, 2.86)***	0.12***	1.90 (1.70, 2.12)***	0.05***	**Age**	1.17 (1.15, 1.19)***	0.14***
											**Education**	0.58 (0.44, 0.78)***	
**AD No Demographics**	1.24 (1.11, 1.39)***	0.00***	1.14 (1.02, 1.27)*	0.00*	1.61 (1.45, 1.79)***	0.03***	1.50 (1.34, 1.68)***	0.02***	1.48 (1.33, 1.65)***	0.02***			
		
**Female**	2.62 (2.34, 2.93)***	0.16***	1.49 (1.32, 1.68)***	0.02***	2.74 (2.43, 3.09)***	0.16***	2.75 (2.44, 3.09)***	0.17***	2.17 (1.93, 2.45)***	0.09***	**Age**	1.19 (1.16, 1.21)***	0.17***
											**Education**	0.79 (0.57, 1.08)	
**Male**	2.17 (1.9, 2.48)***	0.08***	1.25 (1.10, 1.43)***	0.01***	2.36 (2.07, 2.69)***	0.11***	2.28 (1.98, 2.63)***	0.08***	1.66 (1.46, 1.89)***	0.04***	**Age**	1.13 (1.11, 1.16)***	0.10***
											**Education**	0.50 (0.369, 0.687)***	
**≤ 64 Years**	1.74 (1.24, 2.46)**	0.01**	1.30 (1.07, 1.57)*	0.01*	2.24 (1.63, 3.07)***	0.03***	2.01 (1.46, 2.77)***	0.02***	1.61 (1.32, 1.96)***	0.03***	**Age**	1.08 (0.96, 1.22)	0.01**
											**Education**	0.51 (0.33, 0.79)**	
**≥ 65 Years**	2.05 (1.86, 2.27)***	0.09***	1.32 (1.19, 1.47)***	0.01***	2.31 (2.05, 2.59)***	0.09***	2.14 (1.93, 2.39)***	0.09***	1.74 (1.57, 1.92)***	0.05***	**Age**	1.16 (1.13, 1.18)***	0.09***
											**Education**	0.67 (0.52, 0.87)**	
**APOE4 Negative**	2.32 (2.10, 2.56)***	0.12***	1.31 (1.16, 1.48)***	0.01***	2.54 (2.28, 2.83)***	0.14***	2.46 (2.21, 2.74)***	0.13***	2.01 (1.81, 2.24)***	0.07***	**Age**	1.15 (1.13, 1.18)***	0.14***
											**Education**	0.61 (0.46, 0.80)***	
**APOE4 Positive**	2.84 (2.49, 3.37)***	0.15***	1.28 (1.08, 1.51)***	0.01***	2.95 (2.48, 3.52)***	0.16***	2.96 (2.47, 3.53)***	0.15***	1.81 (1.54, 2.12)***	0.05***	**Age**	1.19 (1.15, 1.22)***	0.17***
											**Education**	0.58 (0.40, 0.85)**	

Notes: Logistic Regression models of all dementia risk indices and a simple demographics model across all-case dementia and Alzheimer’s Disease (AD). Stratified analyses have only been performed for all-case dementia. Model significance are indicated as *** *p* < 0.001, ** *p* < 0.005, * *p* < 0.05.

### 3.3 Dementia risk indices without demographics

Excluding demographic variables resulted in a reduction in predictive power across all indices (Table 2). CogDrisk remained the strongest predictor (AUC=0.654), followed by LIBRA (AUC=0.631) and LIBRA2 (AUC=0.628;Table 3). CAIDE had the weakest association (OR=1.17, 95 %CI: 1.08–1.28; AUC=0.547). All indices performed significantly worse than the demographics-only model (all *p* < 0.001, Fig. 1; Table 3.

### 3.4 Alzheimer’s disease and risk indices

All risk indices were associated with AD status (Table 2). CogDrisk (OR=2.55, 95 %CI: 2.29–2.85; AUC=0.759), and LIBRA (OR=2.56, 95 %CI: 2.29–2.86; AUC=0.751) demonstrated the highest predictive performance. LIBRA2 exhibited moderate performance (OR=1.90, 95 %CI: 1.70–2.12; AUC=0.683), while CAIDE had the lowest predictive ability (OR=1.33, 95 %CI: 1.19–1.48; AUC=0.581). The demographics-only model achieved an AUC of 0.774. ANU-ADRI (*p* = 0.026), CAIDE (*p* < 0.001), and LIBRA2 (*p* < 0.001) performed significantly worse than the demographics-only model (Fig. 1). Excluding demographic variables resulted in a reduction in predictive power across all indices, with results closely mirroring those observed for dementia prediction (Table 3).

**Table 3:** Table showing AUC values for each logistic regression model predicting all-case dementia and AD.

Index	N	Demographics	ANU_ADRI	CAIDE	CogDrisk	LIBRA	LIBRA2
**Model**		AUC (95 % CI)	AUC (95 % CI)	AUC (95 % CI)	AUC (95 % CI)	AUC (95 % CI)	AUC (95 % CI)
**Dementia**	5247	0.76 (0.74–0.78)	0.74 (0.72–0.76)**	0.59 (0.56–0.61)***	0.76 (0.74–0.78)	0.75 (0.72–0.77)*	0.69 (0.66–0.71)***
**Dementia No Demographics**	5247	0.76 (0.74–0.78)	0.58 (0.56–0.61)***	0.55 (0.52–0.57)***	0.65 (0.63–0.68)***	0.63 (0.61–0.66)***	0.63 (0.60–0.65)***
**AD**	5009	0.77 (0.75–0.80)	0.75 (0.72–0.78)*	0.58 (0.55–0.61)***	0.76 (0.73–0.79)	0.75 (0.72–0.78)	0.68 (0.65–0.71)***
**AD No Demographics**	5009	0.77 (0.75–0.80)	0.56 (0.53–0.60)***	0.54 (0.51–0.57)***	0.63 (0.60–0.67)***	0.62 (0.59–0.65)***	0.62 (0.59–0.65)***
**Female**	2768	0.78 (0.76–0.81)	0.78 (0.75–0.81)	0.61 (0.58–0.64)***	0.78 (0.75–0.81)	0.78 (0.75–0.81)	0.72 (0.69–0.75)***
**Male**	2479	0.73 (0.70–0.77)	0.69 (0.66–0.73)*	0.57 (0.53–0.60)***	0.74 (0.71–0.77)	0.70 (0.67–0.74)	0.65 (0.61–0.68)***
**≤64 Years**	2569	0.59 (0.53–0.64)	0.60 (0.54–0.65)	0.58 (0.53–0.63)	0.63 (0.57–0.68)	0.63 (0.57–0.68)	0.63 (0.58–0.69)
**≥ 65 Years**	2678	0.71 (0.68–0.74)	0.70 (0.67–0.73)	0.58 (0.55–0.60)***	0.70 (0.67–0.73)	0.70 (0.67–0.73)	0.65 (0.63–0.68)***
**APOE4 Negative**	3729	0.76 (0.73–0.78)	0.73 (0.70–0.76)*	0.57 (0.54–0.60)***	0.76 (0.73–0.79)	0.74 (0.71–0.77)	0.70 (0.67–0.73)***
**APOE4 Positive**	1355	0.79 (0.75–0.82)	0.76 (0.72–0.80)	0.57 (0.53–0.61)***	0.78 (0.74–0.81)	0.76 (0.72–0.80)	0.67 (0.63–0.71)***

Notes: The simple demographics model comprising age and education has been set as the gold standard in a gold standard analysis, *** *p* < 0.001, ** *p* < 0.005, * *p* < 0.05 indicate where indices significantly deviate from the demographics model in predicative ability.

### 3.5 Stratified analysis

When stratified by sex, all indices showed stronger predictive ability in females than males. In females, LIBRA (AUC=0.782), CogDrisk (AUC=0.778), and ANU-ADRI (AUC=0.776) performed similarly to each other (*p* > 0.05) and significantly better than LIBRA2 (AUC=0.715) and CAIDE (AUC=0.608) (*p* < 0.001). In males, CogDrisk (AUC=0.738) significantly outperformed all other indices (*p* < 0.001), while LIBRA2 (AUC=0.648) and CAIDE (AUC=0.566) performed significantly worse than all other indices (*p* < 0.005). Age was a strong predictor in both sexes (AUC=0.784 in females, 0.734 in males), while education was a significant predictor in males (*p* < 0.001) but not in females (*p* = 0.134).

Among age groups, all indices performed better in individuals aged ≥65 at baseline (Fig. 1). In individuals under 65 years at baseline, all indices performed similarly with no significant differences between them (*p* > 0.05, Table 3 and Fig. 1). In older adults (≥65), ANU-ADRI (AUC=0.699), CogDrisk (AUC=0.701), and LIBRA (AUC=0.699) performed similarly (*p* > 0.05), while LIBRA2 (AUC=0.653) and CAIDE (AUC=0.576) were significantly weaker (*p* < 0.001). Between APOE4 carriers, CogDrisk (AUC=0.775), LIBRA (AUC=0.762), and ANU-ADRI (AUC=0.760) performed similarly to each other and the demographics only model (AUC=0.79; *p* > 0.05), while LIBRA2 (AUC=0.668) CAIDE (AUC=0.567) performed significantly weaker (*p* < 0.001). In non-carriers, the same ranking was observed but with slightly lower performance (see Table 2, Table 3).

### 3.6 Sensitivity analysis

Joint multivariate normal imputation was conducted to assess the robustness of the findings. Results remained consistent with the complete case analysis, with all indices retaining their significant associations with dementia. Effect sizes were slightly attenuated, but comparable, with CogDrisk and LIBRA maintaining the highest predictive accuracy. Relative Variance Increase (RVI) values were modest (0.10–0.28, Table 4), indicating that missing data had minimal impact on results. The Fraction of Missing Information (FMI) was highest for LIBRA2 (0.35; Table 4) but remained within an acceptable range across indices. Additionally, an analysis using all available cases without adjusting for missing data produced comparable results, with only minor variations in effect sizes and AUC values (see Table 4)

**Table 4:** Sensitivity analysis using Multiple Imputation, and index-specific complete case analysis.

Sensitivity Analysis with Imputed Data							
Variable	Coeficient	(95 % Conf. Interval)	F-test	Average RVI	Largest FMI	Observations	Imputed Cases
ANU-ADRI	0.91***	(0.84, 0.97)	688.62***	0.10	0.15	8379	2750
CAIDE	0.32***	(0.25, 0.39)	81.98***	0.12	0.19	8379	1488
CogDrisk	0.94***	(0.87, 1.02)	697.83***	0.11	0.16	8379	2805
LIBRA	0.94***	(0.87, 1.01)	660.99***	0.13	0.18	8379	2749
LIBRA2	0.71***	(0.63, 0.79)	297.97***	0.28	0.35	8379	2861

Sensitivity Analysis with Index-Specific Complete Cases							
Variable	Odds Ratio	(95 % Conf Interval)	Pseudo r-squared	Chi2	AIC	ROC	Observations
ANU-ADRI	2.43***	(2.24, 2.63)	0.13	527.92***	1746.07	0.74	5629
CAIDE	1.38***	(1.28, 1.48)	0.02	80.58***	2630.22	0.59	6891
CogDrisk	2.56***	(2.36, 2.78)	0.14	539.34***	1700.92	0.76	5574
LIBRA	2.56***	(2.36, 2.79)	0.14	544.5***	1737.90	0.75	5630
LIBRA2	1.99***	(1.83, 2.16)	0.07	265.21***	1806.53	0.69	5518
Age	1.15***	(1.14, 1.16)	0.14	941.84***	2867.25	0.76	8379
Education	0.64***	(0.54, 0.76)					

Notes: Significance is set as *** *p* < 0.001, ** *p* < 0.005, * *p* < 0.05. Imputed sensitivity analyses using joint multivariate normal imputation (100 datasets; predictors: age, sex, education, dementia status); index-specific complete case sensitivity analyses using all participants with available data for each individual index irrespective of their inclusion in other indices.

## 4 Discussion

This study aimed to compare the predictive accuracy of ANU-ADRI, CAIDE, CogDrisk, LIBRA, and LIBRA2 over an 11-year follow-up in a representative Norwegian population cohort. Their predictive performance was evaluated against a simple demographics model comprising age and education. Our results show that while all indices predicted all-cause dementia and AD statistically significantly, none outperformed the demographics model. Of all indices, CogDrisk emerged as the strongest predictor, demonstrating the highest coefficients and AUC across most analyses and subgroups. Along with LIBRA, it was the only index that did not perform significantly worse than the demographics model. This is in line with previous reports that demographic variables alone often achieve similar discriminative power as any index [16,21,28]. Consistent with previous findings [24,26], CAIDE consistently demonstrated the weakest predictive performance, followed by LIBRA2, which performed significantly worse than the demographics model and CogDrisk in almost all analyses, except in individuals under 65 years at baseline, where no significant differences were observed between indices.

Beyond our findings showing that demographics alone achieve better or similar AUC values to all tested indices, our results, also consistent with previous studies [24], show that removing demographic data greatly diminishes index accuracy. This suggests that age and education account for most of the predictive power within the indices, while other variables contribute little additional accuracy; possibly because they serve as proxies for a broad range of latent exposures or because the indices lack sufficiently refined weighting to capture risk differentials across subgroups.

When stratifying by sex, indices showed stronger predictive accuracy in females than in males. Among females, LIBRA, CogDrisk, and ANU-ADRI performed similarly and significantly better than LIBRA2 and CAIDE. Among males, CogDrisk outperformed all others, and LIBRA2 and CAIDE had the weakest performance. The indices explained 50 %–140 % more variance in dementia risk in females, revealing more pronounced sex disparities than previous studies, which often reported minimal sex differences or better index performance in males [24,46]. Although sex was not independently linked to dementia, stratifying by sex significantly impacted each index’s accuracy, implying that sex not only modulates individual risk factor expression [32,33], but also risk indices overall. These findings further underscore the importance of refining weighting, and future research should explore whether sex differences persist in other cohorts and whether more precise adjustments could improve predictive accuracy.

Predictive accuracy varied across age groups and was generally lower in younger participants (<65), likely due to lower dementia prevalence (4.0 % vs. 17.3 % in older adults), which limits model discrimination. However, it was unexpected that all indices performed equally poorly in this group, with no significant differences between them. CAIDE and LIBRA were designed to capture midlife risk factors [11,12] and were anticipated to outperform indices developed on older populations [14,16]. Notably, CAIDE performed similarly in both age groups, despite prior evidence that it performs worse in older age groups, likely due to it attributing the same weights to everyone over 53 years [11].

Interestingly, LIBRA performed significantly better in the older age group, despite its midlife orientation. LIBRA performed similar to ANU-ADRI and CogDrisk, which were developed with broader age applicability. These indices assign zero weight to BMI and hypertension in those over 60, as those risk factors are known to reverse their association with dementia risk in late life [47,48]. However, this approach is coarse and does not fully capture the complex, age-dependent nature of dementia risk; for instance, none of the indices assign risk to underweight, which is a well-established late-life risk factor [49]. Future research should explore more nuanced, age-stratified weighting reflecting the dynamic relationship between risk factors and dementia across the lifespan. Additionally, longer follow-up periods may be necessary to adequately evaluate risk prediction in younger age groups.

Stratification of APOE4-carries showed the same patterns as the other analyses with CogDrisk, LIBRA, and ANU-ADRI performing similarly, and LIBRA2 and CAIDE showing significantly weaker performance. In non-carriers, the same ranking was observed but with slightly lower performance. This indicates that the predictive performance of the indices is largely consistent irrespective of APOE4 status, and stratification by genetic risk does not significantly enhance their discriminatory ability compared to the unstratified analysis.

### 4.1 Strengths and limitations

This study has several strengths. By using data from 5247 participants in the HUNT4 70+ study, representing over one-quarter of the regional population aged 70+, our findings are based on a large, representative cohort, enhancing external validity. With 566 dementia cases, the study had sufficient power [50] to compare and validate five dementia risk indices across multiple stratifications, including age, sex, and APOE4 status. Additionally, the 11-year follow-up period is as long or longer than in most previous validation studies [21], providing a more comprehensive assessment of long-term dementia risk prediction and reduced likelihood of reverse causality, as the follow-up period exceeds the average duration of the pre-clinical stage of dementia measured at age 70 [51].

The availability of genetic data and linkage to public health registries further strengthened the analysis by allowing for near-complete index construction and supplementation with validated patient information, thereby minimizing missing data. Dementia diagnoses were rigorously established by a multidisciplinary team using DSM-5 criteria, ensuring greater diagnostic accuracy compared to studies relying on self-reports, Mini Mental State Examination scores, or hospital records [13,27]. This study is also the largest to date to provide a direct, side-by-side comparison of ANU-ADRI, CAIDE, CogDrisk, LIBRA, and LIBRA2 under identical conditions. While previous research has validated individual indices or smaller subsets, this study offers an evaluation of all five tools within the same large cohort. Additionally, it is one of the few studies to compare index performance in late mid-life and late life. Sensitivity analyses confirmed the robustness of findings, reinforcing the reliability of index predictions despite missing data.

Despite these strengths, several limitations should be noted. The relatively low dementia prevalence in those who were under 65 years at HUNT3 (4.0 % vs. 17.3 % in those ≥65 at HUNT3) likely contributed to lower AUCs in younger participants, as low case numbers inherently limit model discrimination. While the follow-up period was substantial, it may not fully capture long-term cumulative risk trajectories, particularly for midlife-focused indices. Additionally, several variables, including physical activity, smoking, and social/cognitive engagement, hearing loss, and some medical conditions were self-reported, potentially introducing potential measurement errors. The sensitivity of the thresholds of some of the risk factors was not established, which may have reduced the predictive accuracy. The HUNT dataset also lacks information on pesticide exposure and chronic kidney disease, limiting full validation of ANU-ADRI and LIBRA, which include these factors. Kidney disease, however, is a frequently missing variable [13], making this study comparable to many other LIBRA validation studies. Like other validation studies, this analysis was limited by the availability of variables in the dataset. The original CAIDE, LIBRA, ANU-ADRI, and CogDrisk indices have published questionnaires, but direct evaluation of these instruments is not feasible in most retrospective cohort studies. Instead, we constructed approximations based on available data, reflecting the conceptual structure and intended variable weighting of each index. As such, this study evaluates the statistical implementation of these indices within the HUNT dataset, and the feasibility of their real-life applicability as practical tools in research settings. While the degree of measurement error introduced by these approximations has not been formally assessed, this approach reflects the way these tools are likely to be implemented in retrospective and cohort based research [13,16,[18], [19], [20],[24], [25], [26], [27]].

Our study reports a prevalence of dementia of 10.8 % which is similar to- or better than previous studies in terms of sample representativeness [16,24,27]. Despite this, prevalence in our sample was 26 % lower than general estimates of dementia prevalence in those above 70 years in Norway (14.6 % [38]). While sensitivity analyses suggest that missingness did not substantially impact results, missing data were systematically associated with demographic and health characteristics, introducing the possibility of bias. Prior research found that dementia diagnoses were relatively higher among non-participants [35]. This selective attrition likely led to an underestimation of the associations between risk indices and dementia outcomes, as individuals at higher risk were disproportionately lost to follow-up. This underestimation may have further been amplified by healthy survivor bias and lack of information on competing risks due to death occurring between HUNT3 and HUNT4. Future work should address this more formally using registry-linked mortality and diagnosis data and methods such as inverse probability weighting or competing risk models based on the full HUNT3 cohort. Furthermore, dementia diagnoses were not confirmed via biomarkers, which may introduce some misclassification bias [4,52].

### 4.2 Conclusion

The current study found that, among five commonly used dementia risk indices, CogDrisk and LIBRA performed best, while CAIDE and LIBRA2 showed the weakest predictive ability. However, no dementia risk index outperformed a demographics-only model, underlining the central role of age and education in risk prediction. These findings highlight the need to evaluate whether risk indices provide meaningful improvements over demographics-only models in research settings. Given the extensive validation of the individual risk factors included in all indices, these indices still provide valuable insight for individuals and in clinical settings however, it is unclear how valuable the risk score is beyond providing a checklist of presence or absence of each risk factor. This study underlines that different applications may call for different methods: while demographics-based models are useful for broad risk estimation, indices like CogDrisk and LIBRA remain valuable for risk reduction strategies in clinical and policy settings due to their emphasis on modifiable risk and protective factors. Age- and sex-stratified analyses suggest that refining weighting could improve predictive accuracy, and future research should optimize indices for different age groups and explore possible sex-specific adjustments.

## Funding

This project is funded by the Norwegian Research Council (303,419).

The genotyping was financed by the National Institute of health (NIH), University of Michigan, The Norwegian Research council, and Central Norway Regional Health Authority and the Faculty of Medicine and Health Sciences, Norwegian University of Science and Technology (NTNU). The genotype quality control and imputation has been conducted by the K. G. Jebsen center for genetic epidemiology, Department of public health and nursing, Faculty of medicine and health sciences, Norwegian University of Science and Technology (NTNU). GCF is funded by the Faculty of Medicine and Health Sciences at NTNU and Central Norway Regional Health Authority.

MK was supported by the Wellcome Trust (221,854/Z/20/Z), the UK Medical Research Council (MR/Y014154/1), and the Research Council of Finland (350,426). KJA is funded by ARC Laureate Fellowship FL190100011.

## Data sharing

The data used in this study is owned by the HUNT research center, Statistics Norway, the Norwegian Patient Registry and the Norwegian Cause of Death Registry. Access requires approval from the Regional Ethics Committees and the data owners. The authors are not permitted to share the data with third parties but can be contacted with questions.

## Declaration of generative AI and AI-Assisted technologies in the writing process

During the preparation of this work, the author(s) used ChatGPT (OpenAI) and Microsoft Copilot to provide critical feedback on clarity and language. No AI-generated content was inserted directly, and the author(s) take full responsibility for the content of the publication.

## CRediT authorship contribution statement

**Josephine Stubs:** Writing – review & editing, Writing – original draft, Visualization, Project administration, Methodology, Formal analysis, Data curation, Conceptualization. **Ellen Melbye Langballe:** Writing – review & editing, Supervision, Resources, Project administration, Methodology, Formal analysis, Conceptualization. **Gill Livingston:** Writing – review & editing, Supervision, Funding acquisition, Conceptualization. **Kaarin J. Anstey:** Writing – review & editing, Funding acquisition, Conceptualization. **Kay Deckers:** Writing – review & editing. **Fiona E. Mathews:** Writing – review & editing, Methodology, Funding acquisition, Conceptualization. **Mika Kivimäki:** Writing – review & editing, Funding acquisition, Conceptualization. **Bjørn Heine Strand:** Writing – review & editing, Methodology, Funding acquisition, Conceptualization. **Anne-Marie Rokstad:** Writing – review & editing, Funding acquisition, Conceptualization. **Steinar Krokstad:** Writing – review & editing, Funding acquisition, Conceptualization. **Geir Selbæk:** Writing – review & editing, Supervision, Resources, Project administration, Methodology, Funding acquisition, Formal analysis, Data curation, Conceptualization.

## Declaration of competing interest

The authors declare that they have no known competing financial interests or personal relationships that could have appeared to influence the work reported in this paper.
